# Determination of Quercetin and Resveratrol in Whole Blood—Implications for Bioavailability Studies

**DOI:** 10.3390/molecules15096570

**Published:** 2010-09-20

**Authors:** Lucia Biasutto, Ester Marotta, Spiridione Garbisa, Mario Zoratti, Cristina Paradisi

**Affiliations:** 1 Department of Biomedical Sciences, Università di Padova, viale G. Colombo 3, 35121 Padova, Italy; 2 Department of Chemical Sciences, Università di Padova, via Marzolo 1, 35131 Padova, Italy; 3 CNR Institute of Neuroscience, viale G. Colombo 3, 35121 Padova, Italy

**Keywords:** polyphenols, bioavailability, blood analysis, plasma, quercetin, resveratrol, HPLC

## Abstract

Resveratrol (*trans*-3,4',5-trihydroxystilbene) and quercetin (3,3’,4’,5,7-pentahydroxyflavone) are two naturally occurring polyphenols with the potential to exert beneficial health effects. Since their low bioavailability is a major obstacle to biomedical applications, efforts are being made to improve their absorption and slow down phase II metabolism. An accurate evaluation of the corresponding levels in the bloodstream is important to assess delivery strategies, as well as to verify claims of efficacy based on *in vitro* results. In the present work we have optimized a simple method ensuring complete stabilization and extraction of resveratrol and quercetin from whole blood. The suitability of different protocols was evaluated by measuring the recovery of polyphenol and internal standard from spiked blood samples via HPLC/UV analysis. The optimized procedure ensured a satisfactory recovery of both internal standards and compounds. Comparing plasma and whole blood, up to 76% of the analyte, being associated with the cellular fraction, was unaccounted for when examining only plasma. This indicates the importance of analysing whole blood rather than plasma to avoid underestimating polyphenol absorption in bioavailability studies.

## 1. Introduction

Polyphenols are a vast class of plant-made molecules exhibiting a variety of properties and effects of relevance to many areas of health care [1]. Because of their relative abundance in foods and the potential therapeutic and disease-preventing usefulness of their multiple biological effects quercetin and resveratrol are among the polyphenols attracting most attention [2,3,4,5]. Furthermore, they have long been used, quercetin in particular, in studies dealing with bioavailability, absorption and metabolism. We have therefore chosen them as model compounds.

Many potentially useful effects do not take place *in vivo* to any significant extent because polyphenols are poorly absorbed and rapidly transformed into phase II conjugates, so that circulating levels of unmodified compounds are generally in the 10^-9^-10^-7^ M range, even after an enriched meal. Efforts are under way to modify this situation using a variety of approaches, including pro-drugs, formulations, and macromolecular vectors (liposomes, nanoparticles) [6,7,8,9].

The most practical way to evaluate the outcome of such efforts is via blood analysis. Several methods for the analysis of these polyphenols in different biological samples (plasma, urine, tissues) have been developed [10,11,12,13,14,15,16]. Pharmacokinetic studies have been carried out focusing in most cases on the levels of polyphenols in plasma/serum [17,18,19]. Analysis of plasma, however, does not accurately report overall circulating levels, since quercetin [20,21,22] and resveratrol [23] are known to partition into blood cells, associating with cell membranes, haemoglobin, other proteins and DNA. This bound pool may be considered as a “reserve”, at least partially protected from conjugative enzymes and thus with a potentially extended lifetime, which can replace the molecules eliminated via metabolism and excretion. 

The membrane-associated fraction of absorbed polyphenols is biochemically very relevant, since several known targets of these compounds are membrane components. Their anti-oxidant effects is also partly exerted at the membrane level [22,24]. A proper assessment of absorption/ bioavailability/efficacy ought therefore to take this portion into account, also because in a complex system such as blood the distribution between free and bound populations may not necessarily be constant as the total load varies [25]. Plasma content is not in any case directly related to activity, since polyphenols are well known to bind avidly to albumin and other proteins, and are therefore “sequestered” in plasma as well [26,27,28]. Analysis of whole blood is indeed preferred for other drugs with low solubility in water and high degree of association to blood components, for example propofol [29], methotrexate [30] or cyclosporine-A [31]. 

The aim of the present work was the development of a simple method for resveratrol and quercetin analysis in whole blood and the comparison with the results obtained by analysis of plasma.

## 2. Results and Discussion

### 2.1. Development of the sample treatment protocol

The general protocol developed for sample treatment included acidification with or without the use of an antioxidant (ascorbic acid) to preserve compound stability. The subsequent addition of an excess of organic solvent followed by sonication or vortexing ensured compound extraction and protein denaturation. The sample was finally cleared of cell debris and denatured proteins by centrifugation; the supernatant was collected, concentrated and analyzed by HPLC/UV. Different combinations of organic solvent (ethyl acetate, acetonitrile and acetone) and acid (perchloric acid, citric acid and acetic acid) were tested using resveratrol as model compound. The suitability of the various combinations used was evaluated measuring the recovery percentages of resveratrol and internal standard after the treatment. The acetone/perchloric acid and acetone/acetic acid/ascorbic acid combinations ensured the best recovery for resveratrol, and were therefore also tested with quercetin; the latter prevented oxidation of quercetin and was therefore adopted (see 3.3 for details). 

### 2.2. Method validation

To validate the method, we considered the following parameters: recovery, precision, linearity, selectivity and sensitivity. Absolute recovery was evaluated in samples spiked with an analyte concentration of 5 μM as the ratio between the analyte HPLC/UV peak area in solutions obtained from treated blood samples, normalized to take into account volume changes due to sample treatment (see Section 3.3), and that in a reference aqueous solution, representing 100% recovery. The recovery of the internal standards and the reproducibility of the ratio of recovery of analyte and standard were also determined.

The method ensured a mean recovery of (80 ± 5)% and (87 ± 9)% for resveratrol and quercetin, respectively (means from seven and eight independent treatments, respectively). Recovery of both analytes from spiked samples incubated at 37 ºC for different time periods (5, 30, 45 and 60 minutes before treatment) was similar.

Analytes and internal standards were recovered with a reproducible ratio, equal to 1.18 ± 0.14 (N = 7) for resveratrol/4,4’dihydroxybiphenyl and 0.88 ± 0.06 (N = 8) for quercetin/2’,5,7-trihydroxy-flavone. 

**Figure 1:**
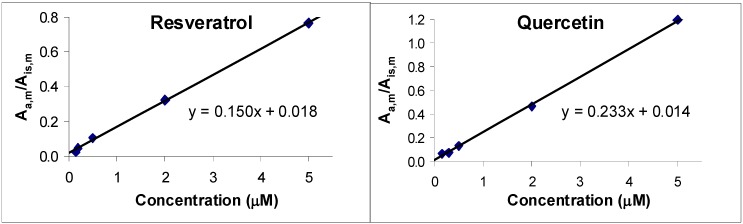
Linear regression of the ratio of the peak area of (a) resveratrol and (b) quercetin (at 320 and 370 nm, respectively) to that of their standards (at 286 and 370 nm, respectively) as a function of the concentration of the former.

Linearity was evaluated by spiking whole blood with different concentrations of resveratrol or quercetin. Plots of the ratio of the peak areas of the analyte and of the internal standard in the blood sample (A_a,m_/A_is,m_) *vs.* analyte concentration (internal standard was always 25 µM) were linear (with a correlation coefficient of 0.9994 and 0.9995 for resveratrol and quercetin, respectively; Figure 1). These plots confirm proportionality between A_a,m_ and actual analyte concentration and the constancy of the ratio of recovery of analyte and standard.

The precision of the analytical method was determined analyzing 3 aliquots of a sample of blood spiked with resveratrol or quercetin. The precision, expressed as coefficient of variation, was 1.6% for resveratrol, 2.7% for quercetin, 1.1% for 4,4’-dihydroxybiphenyl and 3.1% for 2’,5,7-trihydroxy-flavone. 

**Figure 2:**
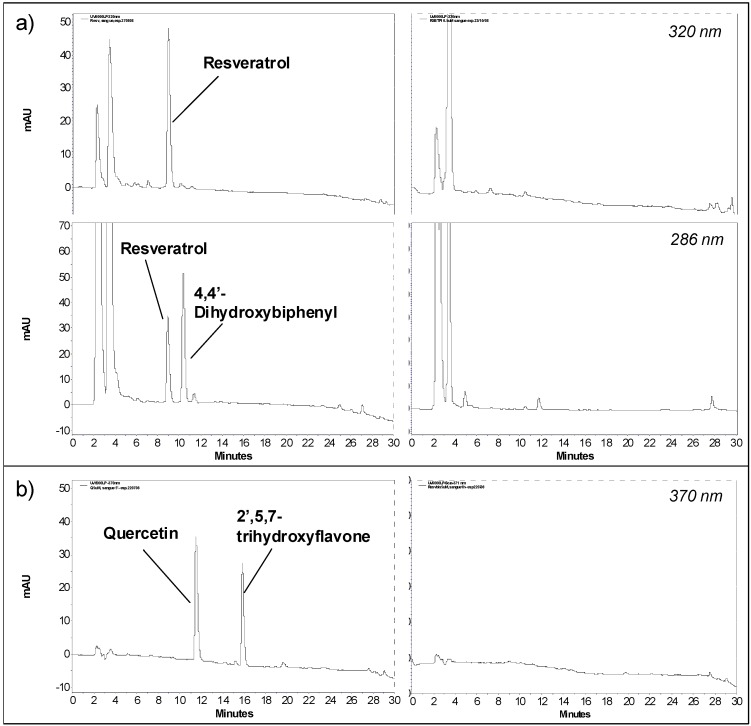
HPLC chromatograms of treated blood samples spiked with (a) 5 μM resveratrol (recorded both at 286 and 320 nm) and (b) 5 μM quercetin (recorded at 370 nm). Panels at right show the HPLC chromatogram (at the same wavelength) of a treated blood sample without any spiking.

To evaluate the selectivity of the method resveratrol or quercetin, together with their internal standards, were quantified at 320 nm (resveratrol), 286 nm (4,4’-dihydroxybiphenyl) and 370 nm (quercetin and 2’,5,7-trihydroxyflavone). The chromatograms at these wavelengths show that both analytes and their internal standards are well resolved and free from interfering blood matrix background peaks (Figure 2).

Limit of detection (LD) and limit of quantification (LQ) of resveratrol and quercetin were calculated measuring the average background response at 320 and 370 nm, respectively, in 10 different HPLC runs. LD and LQ were taken to be 3 and 10 times, respectively, the standard deviation of this background response, according to the definition accepted by both IUPAC and the American Chemical Society. Interpolating the data obtained with calibration curves in H_2_O:CH_3_CN for resveratrol and quercetin, we obtained an LD of 0.04 µM and an LQ of 0.12 µM for resveratrol and an LD of 0.05 µM and an LQ of 0.17 µM for quercetin. LQ was confirmed analyzing a solution at the LQ concentration of resveratrol and quercetin; peak area of both the analytes at the LQ concentration fit well within the calibration curve. It should be noted that these parameters refer to the actual solution being analysed. If analytes are present in the original blood samples at concentrations below LQ, they can be brought above the LQ by the simple expedient of concentrating the extract. 

### 2.3. Analysis of whole blood vs. analysis of plasma

**Figure 3:**
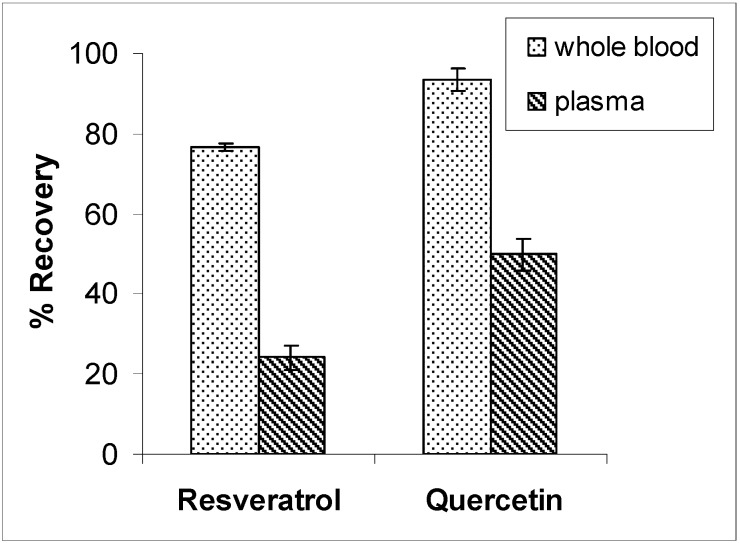
Mean recoveries of resveratrol and quercetin from whole blood and plasma, coming from samples spiked with 5 μM quercetin or resveratrol (N = 3).

We analyzed samples of whole blood and plasma obtained from the same spiked blood samples. In plasma the recoveries of resveratrol and quercetin were (24 ± 3)% and (50 ± 4)%, respectively, while the remainder was retained in the cell pellet obtained upon plasma separation (Figure 3). The comparison with whole blood analysis highlights an incomplete recovery from plasma and indicates that treatment of whole blood rather than plasma is necessary to avoid underestimating the compounds in the circulatory stream. The same results were obtained incubating spiked blood samples for different times (15, 30 or 45 minutes) before separating plasma (data not shown).

## 3. Experimental

### 3.1. Instruments

HPLC/UV analyses were performed on a Thermo Separation Products Inc. system equipped with a P2000 Spectra System pump and a UV6000LP diode array detector (190-500 nm). 

### 3.2. Chemicals

Materials were purchased from Sigma/Aldrich/Fluka/Riedel de-Haen, Merk-Novabiochem, and were used as received. Resveratrol was purchased from Waseta Int. Trading Co. (Shangai, P.R.China) and 2’,5,7-trihydroxyflavone from Indofine (Hillsborough, NJ, USA). 

### 3.3. Preparation of samples

For each set of determinations, one or two Wistar rats from the stabulary of the Dept. of Biomedical Sciences were anesthetized and blood was withdrawn from the jugular vein, heparinized and transferred to tubes containing EDTA. Blood samples (1 mL) were spiked with resveratrol or quercetin (dilution from 1000× stock solutions in DMSO, 0.1% final DMSO). Aliquots (200 μL) were taken and processed to establish the suitability of the method for the analysis of small volumes, such as blood samples collected from rats during pharmacokinetic studies. Before starting the treatment, internal standard was added (4,4’-dihydroxybiphenyl and 2’,5,7-tri-hydroxyflavone, respectively, for resveratrol and quercetin; dilution from 50x stock solutions in methanol, 25 μM final concentration). In the protocol eventually adopted, blood was then stabilized with a freshly-prepared 10 mM solution of ascorbic acid (0.1 vol) and acidified with 0.6 M acetic acid (0.1 vol); after mixing, an excess of acetone (4 vol) was added, followed by sonication (2 min) and centrifugation (10,000 g, 8 min, 4 ºC). An accurately measured portion of the supernatant was finally collected and stored at -20 ºC. Before analysis, acetone was allowed to evaporate at R.T. under N_2_ flow or by using a rotational vacuum concentrator (Martin Christ, RVC 2-25), and CH_3_CN (30 μL) was added to precipitate residual proteins; after centrifugation, cleared samples were directly subjected to HPLC/UV analysis. The residual aqueous solution was not concentrated further. Concentration of this phase may be used to quantify blood levels below the LQ limit, which applies to the solution injected into the HPLC apparatus.

Analyte concentration in the spiked blood samples was 0.15, 0.2, 0.5, 2.0 and 5.0 μM. Internal standard was added at the same concentration (25 µM) in all samples. This range is in line with the concentrations found (in plasma) in several pharmacokinetics animal and human studies which report values ranging from 0 to the micromolar range (e.g., [32,33,34,35,36,37]). Spiked samples were also incubated at 37 ºC for different time periods (5, 15, 45 and 60 minutes) before initiating treatment, to verify possible effects of incubation time on recovery [38]. 

### 3.4. Comparison of plasma and whole blood

Blood samples spiked with 5 µM resveratrol or quercetin and internal standard were incubated at 37 ºC for 15, 30 or 45 minutes. Each sample was then split into two equal aliquots before treatment, and plasma was obtained from one of these by centrifugation (500 g, 10 min, R.T.). Whole blood, plasma and the precipitated blood cells were then treated as described above.

### 3.5. HPLC/UV analysis

Samples (20 μL) were analyzed using a reverse phase column (Gemini C18, 3 μm, 150 × 4.6 mm i.d.; Phenomenex). Solvents A and B were H_2_O containing 0.1% TFA and CH_3_CN, respectively. The gradient for B was as follows: 30% for 5 min, from 30% to 60% in 15 min, from 60% to 100% in 5 min. The flow rate was 0.7 mL/min. The eluate was preferentially monitored at 286 nm for 4,4’-dihydroxybiphenyl, 320 nm for resveratrol, and 370 nm for quercetin and 2’,5,7-trihydroxyflavone.

### 3.6. Calibration/standard curves

Standard solutions of resveratrol or quercetin (0.15, 0.2, 0.5, 1.0, 5.0 and 25 µM in H_2_O:CH_3_CN 7:3) were analyzed by HPLC/UV as described above; peak area (at λ = 320 and 370 nm, for resveratrol and quercetin, respectively) was plotted against concentration to establish the correlation between peak area and amount analyzed.

## 4. Conclusions

The analytical method developed in this work allows the determination of quercetin and resveratrol in whole blood. This constitutes a significant methodological advance, since blood levels appear to be a more appropriate parameter than plasma levels when evaluating overall absorption and bioavailability of polyphenols, as the majority of the molecules in the bloodstream are bound to the cellular component. This is particularly relevant if two or more such compounds are to be compared, since they may be expected to bind to blood constituents to different extents. Considering the widely different molecular structure of the two model polyphenols used, belonging respectively to the stilbene and flavonoid sub-families, it is expected that the developed protocol will also be suitable for other classes of polyphenols. 
